# A Case Report of Continuous Flumazenil Infusion in a Nonagenarian With Benzodiazepine Toxicity, Managed Outside Intensive Care

**DOI:** 10.7759/cureus.88498

**Published:** 2025-07-22

**Authors:** Daniele Romanello, Pietro Pierini, Claudia Brigato, Eugenio Cunego, Sara Rotunno

**Affiliations:** 1 Internal Medicine, Ospedale San Pietro Fatebenefratelli, Rome, ITA; 2 Pharmacy, Ospedale Buccheri La Ferla Fatebenefratelli, Palermo, ITA; 3 Hospital Management, Ospedale San Pietro Fatebenefratelli, Rome, ITA

**Keywords:** benzodiazepine poisoning, benzodiazepine use in delirium, critically ill elderly patients, elderly patient care, flumazenil

## Abstract

Benzodiazepine overdose in older adults with renal impairment can lead to prolonged coma and respiratory depression. Flumazenil is an available antidote, though its use in continuous infusion is rarely documented, especially in geriatric patients outside intensive care. We report the case of a 93-year-old man with chronic kidney disease and acquired solitary kidney who was admitted for acute respiratory failure secondary to aspiration pneumonia. During hospitalization, the patient developed a profound and persistent alteration of consciousness with stertorous breathing and hypotension. After a positive response to a test dose of flumazenil, a continuous infusion was started, resulting in full neurological recovery. The infusion was tapered over 72 hours without significant adverse effects. Unfortunately, the patient died as a result of sepsis caused by *Enterococcus faecalis*. Continuous flumazenil infusion may be a valuable therapeutic strategy in selected elderly patients with suspected benzodiazepine intoxication, allowing resolution of sedation while managing concurrent acute illnesses.

## Introduction

Benzodiazepine overdose is a common clinical condition, particularly significant in older adults. Age-related physiological changes, such as decreased renal and hepatic function, may prolong drug half-life and enhance sedative effects. In this context, even therapeutic doses of benzodiazepines can lead to toxic accumulation, resulting in central nervous system (CNS) and respiratory depression [1,2]. In elderly patients, benzodiazepine intoxication can lead to serious complications. Comatose states make oral therapy administration impossible and, if not properly managed, may result in dehydration. In less severe cases, with partial impairment of consciousness, there is a risk of aspiration of food or liquids. Additionally, prolonged hospitalization may occur, and in more severe cases, respiratory suppression may develop [2-4]. Flumazenil is a competitive antagonist at the gamma-aminobutyric acid (GABA(A)) receptor that rapidly reverses the sedative effects of benzodiazepines. It is typically administered as an intravenous bolus in acute intoxications. However, its short half-life (approximately 40-80 minutes) may be insufficient in cases involving long-acting benzodiazepines or impaired renal function. In such scenarios, continuous flumazenil infusion has been proposed as a therapeutic alternative, although this method is seldom documented and primarily used in intensive care units (ICUs) [2,3]. The literature has highlighted potential risks associated with continuous infusion, including withdrawal symptoms and seizures, especially in patients with chronic benzodiazepine use. Nonetheless, in selected cases without seizure history and with suspected acute overdose, continuous administration can be both effective and safe [4-8]. Despite this, there is limited data regarding the use of continuous flumazenil infusion in elderly, frail patients managed outside of high-intensity care settings. 

In this article, we report the case of a 93-year-old man with advanced chronic kidney disease who presented with deep sedation, likely due to benzodiazepine toxicity. He was successfully treated with continuous flumazenil infusion in a general medical ward, without access to intensive care. This case illustrates the feasibility and potential effectiveness of this strategy in an unconventional clinical setting.

## Case presentation

A 93-year-old man with stage 4 chronic kidney disease in acquired solitary kidney, hypertensive heart disease with chronic heart failure at preserved ejection fraction (HFpEF), atrial fibrillation, was transferred to our unit for severe impairment of consciousness (Glasgow Coma Scale (GCS) = 6). He had been hospitalized elsewhere for aspiration pneumonia, treated with intravenous (IV) piperacillin/tazobactam and corticosteroids. The patient’s family decided to discontinue hospitalization at that facility and opted for transfer to ours.

**Figure 1:**
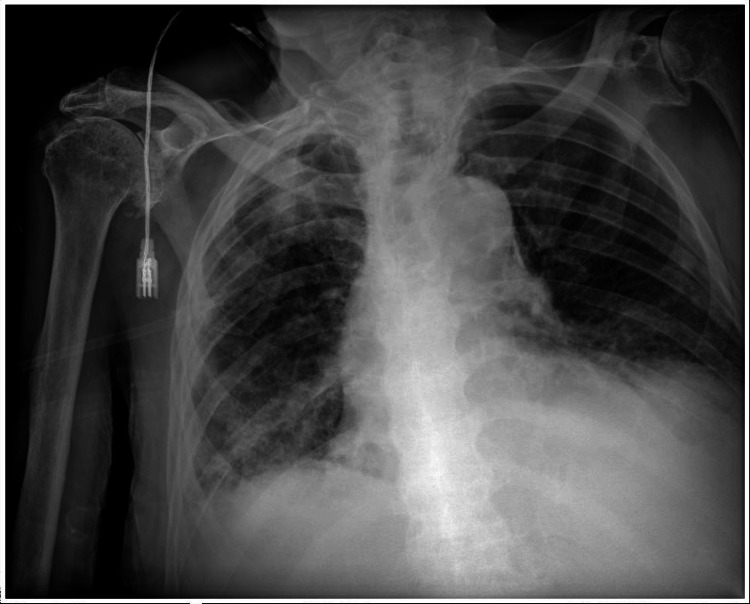
Chest radiography Areas of hypotransparency in the bilateral basal and left retrocardiac regions are consistent with inflammatory-infectious etiology, along with a bilateral basal pleural effusion.

At admission, he was stuporous with stertorous breathing, blood pressure of 70/40 mmHg, heart rate of 100 bpm, and peripheral oxygen saturation of 92% on a 4 L/min nasal cannula. Laboratory tests revealed severe renal failure (according to the RIFLE (risk, injury, failure, loss, and end-stage kidney disease) criteria, this corresponds to stage F - failure), without electrolyte disturbances, and with elevated C-reactive protein (CRP) levels. Blood gas analysis revealed no metabolic acidosis (Table 1). A chest radiography revealed bilateral basal pneumonia with pleural effusion (Figure 1).

**Table 1:** Biochemical value Principal biochemical values and arterial blood gas analysis revealed acute kidney impairment and elevated C-reactive protein (CRP) levels. No electrolyte disturbances or acid-base abnormalities were identified that could account for the comatose state.

Test	Value (range)
Creatinine	5.8 mg/dL (0.67-1.17)
Blood urea nitrogen (BUN)	240 mg/dL (15-50)
Sodium	137 mEq/L (135-148)
Potassium	4.6 mEq/L (3.5-5.1)
C-reactive protein	50.6 mg/L (0.1-5.0)
pH	7.43 (7.38-7.42)
pCO_2_	34 mmHg (35-45)
pO_2_	83 mmHg (80-100)
HCO_3_-	22 mEq/L (21-28)

Despite initial fluid and bicarbonate resuscitation, his neurological condition did not improve even though vital signs had normalized. In the differential diagnosis, metabolic encephalopathy was considered but seemed unlikely, as serum creatinine and blood urea nitrogen (BUN) levels, although elevated, had not increased significantly enough to explain the onset of coma. Hypoxic-ischemic encephalopathy was also considered unlikely given the absence of severe hypoxemia. To rule out central nervous system infection and structural brain lesions, brain imaging would have been appropriate; however, due to the patient’s advanced age and critical condition, it was decided to first evaluate the possibility of sedative overdose. Moreover, the patient’s family reported nocturnal agitation on the previous night, followed by a sudden onset of unresponsiveness, raising suspicion of recent sedative administration. There was no reported history of chronic benzodiazepine use. A test dose of intravenous flumazenil (0.5 mg/mL, 5 mL) was administered. No significant concern was raised regarding potential side effects from flumazenil administration, as the dose initially used was low and intended solely for diagnostic testing. Moreover, the patient had no reported history of chronic benzodiazepine use, which minimized the risk of withdrawal symptoms or seizures. The test resulted in a prompt restoration of consciousness (GCS = 15), supporting the diagnosis of benzodiazepine-induced central nervous system depression [3]. Unfortunately, symptoms recurred within 15 minutes, consistent with flumazenil’s short duration of action. A continuous infusion of 25 mL of 0.5 mg/mL of flumazenil in 250 mL of 0.9% saline solution at 20 mL/hour was started. The patient maintained consciousness and completed antibiotic and fluid therapy. The infusion was gradually tapered and discontinued after 72 hours without any adverse events. In the absence of established literature on the tapering of continuous intravenous flumazenil, dose reduction was guided by clinical monitoring of the patient's level of consciousness, adjusting the infusion rate in steps of 5 mL/min to maintain a GCS score of 15. The clinical condition progressively improved, and the patient remained alert and compliant with the therapeutic plan. Unfortunately, a sepsis caused by *Enterococcus faecalis *gradually worsened the case, ultimately leading to the patient’s death.

## Discussion

Continuous flumazenil infusion is rarely used outside of ICUs due to the risk of withdrawal symptoms, agitation, and seizures, particularly in patients with chronic benzodiazepine use [1,2]. However, in elderly patients with reduced renal clearance, benzodiazepines may accumulate to toxic levels, leading to prolonged central nervous system depression that cannot be reversed by a single bolus dose [3,4].

In this case, the patient was managed entirely in a general medical ward, without access to intensive or sub-intensive care settings. Despite this, continuous flumazenil infusion was safely implemented with strict clinical monitoring and without adverse events. The absence of prior seizure history, the availability of a dedicated caregiver, and a cautious tapering protocol allowed safe administration of the antidote in a non-critical care setting. The patient’s neurological status was closely monitored throughout the flumazenil infusion. The level of consciousness was assessed using the GCS every hour during daytime and every six hours overnight. This approach was adopted due to the unavailability of cardiac telemetry or other continuous monitoring systems, ensuring clinical vigilance through regular bedside assessments. Tapering was performed by reducing the infusion rate by 5 mL/hour every six hours, with clinical assessment during the following hour to evaluate the effect of the reduction. If the GCS remained stable, the new rate was maintained; otherwise, the previous infusion rate was reinstated.

The use of a test dose served both diagnostic and therapeutic purposes, supporting the diagnosis of benzodiazepine-induced encephalopathy and guiding the decision to initiate continuous infusion [3]. The positive clinical response made serum or urinary benzodiazepine testing unnecessary. The restoration of consciousness enabled timely continuation of antibiotic therapy, rehydration, and re-initiation of enteral nutrition, significantly improving the patient's prognosis and hospital outcome.

This case supports the hypothesis that, in selected geriatric patients with high suspicion of benzodiazepine toxicity and no contraindications, continuous flumazenil infusion may be considered outside intensive care, provided that appropriate clinical supervision is ensured. To our knowledge, there are few published reports describing this approach in patients of such advanced age and clinical frailty.

## Conclusions

This case highlights the potential role of continuous flumazenil infusion in the management of acute benzodiazepine toxicity in elderly patients with renal impairment, even when treated outside of intensive care settings. Careful patient selection, close clinical monitoring, and caregiver support were key to the safe and effective use of this therapy in a standard medical ward. In such contexts, continuous infusion may serve not only to reverse sedation but also to support the treatment of concurrent acute illnesses. Further clinical experience and studies are needed to better define safety criteria and monitoring protocols in non-ICU settings.
